# Updates in the Management of Hyperglycemic Crisis

**DOI:** 10.3389/fcdhc.2021.820728

**Published:** 2022-02-09

**Authors:** Mohammed Aldhaeefi, Namareq F. Aldardeer, Nada Alkhani, Shatha Mohammed Alqarni, Abdullah M. Alhammad, Abdulrahman I. Alshaya

**Affiliations:** ^1^ Department of Pharmacy Practice, College of Pharmacy, King Saud bin Abdulaziz University for Health Sciences, Riyadh, Saudi Arabia; ^2^ King Abdullah International Medical Research Center, Riyadh, Saudi Arabia; ^3^ Department of Pharmaceutical Care Services, King Abdulaziz Medical City, Riyadh, Saudi Arabia; ^4^ Department of Pharmacy Services, King Faisal Specialist Hospital & Research Center, Jeddah, Saudi Arabia; ^5^ Department of Pharmacy Services, King Fahad Medical City, Riyadh, Saudi Arabia; ^6^ Doctor of Pharmacy Program, King Saud bin Abdulaziz University for Health Sciences, Riyadh, Saudi Arabia; ^7^ Department of Clinical Pharmacy, College of Pharmacy, King Saud University, Riyadh, Saudi Arabia; ^8^ Department of Pharmacy Services, King Saud University Medical City, Riyadh, Saudi Arabia

**Keywords:** diabetic ketoacidosis, hyperosmolar hyperglycemic syndrome, hyperglycemia crisis, hyperglycemic emergencies, diabetes mellitus

## Abstract

Diabetes mellitus (DM) affects the metabolism of primary macronutrients such as proteins, fats, and carbohydrates. Due to the high prevalence of DM, emergency admissions for hyperglycemic crisis, diabetic ketoacidosis (DKA) and hyperglycemic hyperosmolar state (HHS) are fairly common and represent very challenging clinical management in practice. DKA and HHS are associated with high mortality rates if left not treated. The mortality rate for patients with DKA is < 1% and ~ 15% for HHS. DKA and HHS have similar pathophysiology with some few differences. HHS pathophysiology is not fully understood. However, an absolute or relative effective insulin concentration reduction and increased in catecholamines, cortisol, glucagon, and growth hormones represent the mainstay behind DKA pathophysiology. Reviewing the patient’s history to identify and modify any modifiable precipitating factors is crucial to prevent future events. The aim of this review article is to provide a review of the DKA, and HHS management based on the most recently published evidence and to provide suggested management pathway of DKA of HHS management in practice.

## Introduction

Diabetes mellitus (DM) is a chronic metabolic disorder that disrupts the metabolism of primary macronutrients such as proteins, fats, and carbohydrates (1, 2). DM is a well-known risk factor for cardiovascular disease and increases the mortality rate by 2‐ to 4‐fold (1, 3). DM remains a leading cause of death worldwide and is the number one cause of kidney failure, lower-limb amputations, and adult blindness (1, 3, 4). The global prevalence of DM in 2019 was around 9.3% (463 million people), and this prevalence is expected to be as high as 10.9% (700 million) by 2045 (3). Due to this high prevalence of DM, emergency admissions for hyperglycemic crisis, Diabetic Ketoacidosis (DKA) and Hyperglycemic Hyperosmolar State (HHS), still very common and challenging (1–3). Both conditions have high mortality rates if kept not treated. The mortality rate for patients with DKA is < 1% and ~ 15% for HHS (1). However, higher mortality rates were reported among elderly patients diagnosed with DKA (1).

## Pathophysiology

DKA and HHS have similar pathophysiology with some differences. The pathogenesis behind HHS is not as well understood (2, 5). DKA is a complex metabolic disorder caused by an absolute or relative effective insulin concentration reduction and increased in catecholamines, cortisol, glucagon, and growth hormones (5, 6). Hyperglycemia is explained by three main mechanisms: increased gluconeogenesis, accelerated glycogenolysis, and impaired glucose utilization by peripheral tissues (7). Insulin reduction and increased counterregulatory hormones in DKA accelerate the lipolysis, which results in the release of free fatty acids into the circulation from adipose tissue and stimulates the conversion of fatty acid to ketone by liver oxidation (7, 8). This profound increase in free fatty acid and ketone concentrations lead to a further increase in the magnitude of hyperglycemia by inducing insulin resistance and ultimately results in ketonemia and metabolic acidosis (7, 8).

Previous studies have shown that excessive glucose levels and fatty acids are associated with a pro-inflammatory and oxidative state among DKA patients (9, 10). Oxidative stress is defined as an increase in reactive oxygen species (ROS) generation (9). Overproduction of ROS results in cellular damage of lipids, membranes, and proteins (9). Additionally, the oxidative state increases the risk of developing chronic diabetic complications following the DKA event (9). Significant increase of IL-6, -1B and -8, and TNF-α and other cytokines reduce the response to insulin therapy. Insulin therapy and hydration are essential in normalizing these parameters (9).

In contrast to DKA, insulin production is not significantly reduced among HHS patients (4). Thus, HHS patients present with mild/moderate ketonemia and acidemia. This minimal insulin production is adequate to prevent lipolysis and ketogenesis (4, 5). HHS is characterized by severe elevations in serum glucose concentrations and hyperosmolality (4, 5). This extreme elevation in serum hyperosmolality results in osmotic diuresis, a greater degree of dehydration, and more fluid loss than DKA (4, 5). This significant loss of intracellular fluids results in much higher blood glucose (BG) with HHS in comparison to DKA (4, 5).

Euglycemic DKA is another unique presentation of DKA and has been reported more often recently (6, 11). The exact pathophysiology of euglycemic DKA is not well established; however, the clinical presentation is similar to DKA with a BG of < 250 mg/dl (6, 11). Euglycemic DKA has been linked with many factors, such as treatment of diabetes, carbohydrate restriction, high alcohol intake, and inhibition of gluconeogenesis (6, 11). It also can be induced due to certain medications, most commonly seen with sodium-glucose cotransporter 2 (SGLT-2) inhibitors and insulin (6, 11).

## Diagnosis

### Signs and Symptoms

DKA develops more rapidly in comparison to HHS. In some cases, it only takes a few hours from the precipitating factor for DKA to develop (12). Both metabolic disorders present with classical hyperglycemia symptoms: polyuria, polydipsia, weakness, and mental status changes (6, 12). Moreover, fruity odor to the patient’s breath might be noted. Additionally, patients with HHS and DKA often present with signs of dehydration, such as dry mucous membranes, poor skin turgor, tachycardia, hypotension, and increased capillary refill with severe dehydration (8, 12). If DKA worsens and is left without treatment, it can eventually lead to unconsciousness (6).

### Laboratory Findings

The initial laboratory assessment of patients with suspected DKA or HHS should include BG, blood urea nitrogen, serum creatinine, serum ketones, electrolytes, anion gap, osmolality, urine ketones, and arterial blood gases (6, 8). Assessing the patient’s HbA1c differentiate chronic hyperglycemia of uncontrolled diabetes from acute metabolic decompensation in a previously well-controlled diabetic patient (6). DKA severity is classified as mild, moderate, or severe based on the degree of acidosis and the patient’s mental status. The distinguish diagnostic criterion for DKA is an elevation in circulating total blood ketone and high anion gap metabolic acidosis defined as >12 (4, 6). Other reasons for high anion gap metabolic acidosis, such as ethyl glycol toxicity, isoniazid overdose, lactic acidosis, methanol toxicity, propylene glycol ingestion, salicylates toxicity, and uremia, must be ruled out (13). Majority of patients with DKA present with blood glucose > 250 mg/dL, bicarbonate between 10-15, and elevated anion gap metabolic acidosis > 12 (4, 6). Diagnostic criteria for DKA and HHS are listed in **Table 1** (6). Occasionally, patients with HHS present with mild acidosis (pH >7.30 and bicarbonate level >20 mEq/L) with negative plasma and urine ketone test (4). Patients with a higher level of osmolarity and pH present with worse dehydration and mental status (4). A key diagnostic criteria that differentiate HHS from DKA is the severe elevation in serum osmolality among HHS patients (> 320 mOsm/kg) (4, 6).

**Table 1:** Diagnostic criteria for DKA and HHS.

	DKA			HHS
	Mild (plasma glucose > 250 mg/dl)	Moderate (plasma glucose > 250 mg/dl)	Severe (plasma glucose > 250 mg/dl)	Plasma glucose > 600 mg/dl
**Arterial pH**	7.25–7.30	7.00 to < 7.24	<7.00	>7.30
**Serum bicarbonate (mEq/l)**	15–18	10 to < 15	<10	>18
**Urine ketone**	Positive	Positive	Positive	Small
**Serum ketone**	Positive	Positive	Positive	Small
**Effective serum osmolality**	Variable	Variable	Variable	>320 mOsm/kg
**Anion gap**	>10	>12	>12	Variable
**Mental status**	Alert	Alert/drowsy	Stupor/coma	Stupor/coma

## Treatment and Therapeutics

**Figure 1:**
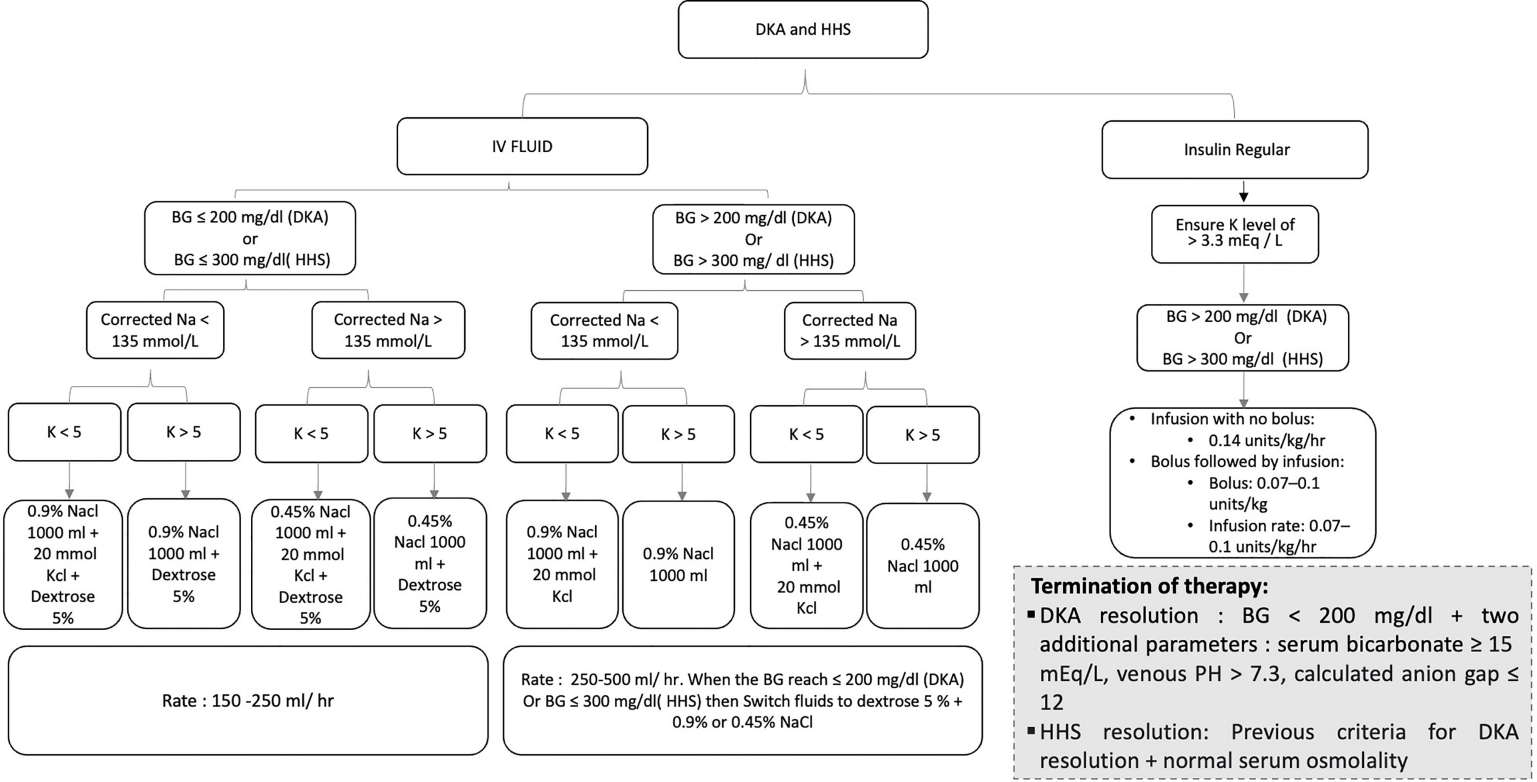
Pathway displays the management of diabetic ketoacidosis (DKA) and hyperglycemic hyperosmolar state (HHS).

DKA resolution is achieved following the correction of dehydration, hyperglycemia, and electrolyte imbalances (2, 6, 8). BG should be < 200 mg/dl, and two additional parameters of the followings must be attained: a serum bicarbonate level ≥ 15 mEq/l, a venous pH > 7.3, and a calculated anion gap ≤ 12 mEq/l (6, 8). In addition to the previously mentioned criteria, normal osmolality is required for HHS resolution (6, 8). Reviewing the patient’s history to identify and modify any modifiable precipitating factors is crucial to prevent future events (6). Numerous DKA/HHS cases can be prevented with appropriate patient education and access to chronic diabetes medications (6). **Figure 1** displays a suggested management pathway of DKA and HHS based on the American Diabetes Association (ADA) 2009 guidelines and Joint British Diabetes Societies for Inpatient Care (JBDS-IP) 2021 revised guidelines (1, 14).

## Fluids Management and Stewardship

Fluid therapy is a cornerstone for the management of DKA and HHS. Aggressive repletion with isotonic saline expands the extracellular volume and stabilizes cardiovascular functions (15). This also has a role in reducing BG (up to 80%) within the first hours of rehydration, reversing ketosis and decreasing serum osmolality therefore increase insulin sensitivity (16).

The initial fluid management general practice and protocols are based on the ADA guidelines 2009 statement for the management of hyperglycemic crises in adult patients with diabetes (1). It recommends initiating 0.9% sodium chloride IV bolus to be infused at a rate of 15-20 mL/kg per hour, around 1 to 1.5 L/hour, in a hypovolemic patient for hemodynamic resuscitation (1). After two to three hours of initial fluid replacement, the rate and type of fluid will depend on corrected sodium concentration (corrected sodium= measured sodium + [1.6 (glucose–100)/100]), serum glucose level, and patient’s volume status. Half normal saline (0.45% sodium chloride) or 0.9% sodium chloride can be subsequently used depending on corrected serum sodium at a rate of 250 to 500 mL/hr (1, 17).

During fluid replacement, it is expected that hyperglycemia will be corrected faster than ketoacidosis and DKA resolution (1). Once serum glucose reaches around 200 mg/dL, the intravenous fluid should be switched to 5% dextrose with 0.45% sodium chloride (1). Appropriate assessment of serum osmolality, urine output, and cardiac function should be performed to guide the aggressive fluid administration and avoid iatrogenic overload (1). These recommendations and DKA/HHS managing practices are guided by expert opinion and tracer studies that evaluated the use of fluid repletion in DKA patients. However, optimal initial fluid therapy for managing DKA or HHS was not evident by clinical trials to evaluate the efficacy and safety outcomes of using normal saline or other crystalloid (1).

It is known that using 0.9% sodium chloride for fluid resuscitation will cause hyperchloremic acidosis (HA) as a side effect of its high content of chloride ions (18). Some practitioners may use balanced fluids as an alternative to overcome this side effect, as its different composition could physiologically lead to a faster resolution of acidosis (18). Common types of crystalloid IV fluids and their composition are listed in **Table 2** (19).

**Table 2:** Composition of common crystalloid IV fluids.

	pH	Na^+^ (mEq/L)	Cl^–^ (mEq/L)	[Na^+^]:[Cl^–^] ratio	K^+^ (mEq/L)	HCO3 ^–^/Bicarbonate	Ca^2+^ (mEq/L)	Osmolarity (mOsm/L)
**0.9% sodium chloride**	5.0	154	154	1:1	0	0	0	308
**0.45 sodium chloride**	4.0	77	77	1:1	0	0	0	154
**Lactated Ringer’s**	6.5	130	109	1.19:1	4	28 (lactate)	3	275
**Isolyte solution**	7.4	140	98	1.43:1	5	27 (acetate)	0	294
						23 (gluconate)		

Small trials evaluated the effect of balanced fluids and 0.9% sodium chloride in managing DKA adult patients suggested that balanced fluids may increase insulin sensitivity or lead to a faster bicarbonate correction and resolution of acidosis (20, 21). A clinical researchers published a post–hoc secondary analysis of previously reported trials, Saline Against Lactated Ringer’s or Plasma-Lyte in the Emergency Department (SALT-ED) and the Isotonic Solutions and Major Adverse Renal Events Trial (SMART), to evaluate the clinical outcome difference between balanced crystalloids and normal saline in DKA patients (22). They found that balanced crystalloids significantly resulted with a shorter median time for DKA resolution than saline (13.0 vs. 16.9 hours, respectively; 95% CI, 1.18-2.38; P = .004) (22). At the same time, it significantly led to a shorter median time for insulin discontinuation than saline (9.8 vs. 13.4 hours, respectively; 95% CI, 1.03-2.03; P = 0.03) (22). A recently published trial, Sodium Chloride or Plasmalyte−148 Evaluation in Severe Diabetic Ketoacidosis (SCOPE−DKA), a cluster, crossover, randomized, controlled trial evaluated the use of Plasmalyte-148 (PL) and 0.9% sodium chloride (SC) at seven Australian hospitals for the management of severe DKA in the intensive care unit (ICU) (23). They found no significant difference in DKA resolution at 48 hours, ICU, and hospital length of stay. However, PL group had significantly reached more DKA resolution at 24 hours in comparison to 0.9% sodium chloride (69% [PL] and 36% [SC], 95% CI 1.68–10.72, p = 0.002) (23).

In conclusion, designing an appropriate fluid repletion therapy for DKA and HHS management will need careful planning and monitoring for choosing the appropriate fluid type, volume, and rate for the patient.

## Insulin Dosing in Diabetic Ketoacidosis

Insulin is considered to be one of the three fundamental elements of DKA and HHS management **(** 2, 6, 24 **).** It reduces hepatic glucose synthesis, enhances peripheral glucose utilization, and inhibits lipolysis, ketogenesis, and glucagon secretion, lowering plasma glucose levels and decreasing ketone bodies production (6, 24). Insulin should be given immediately after the initial fluid resuscitation (2, 6, 24). The aim of using insulin in DKA and HHS is to close the anion gap generated by the production of ketone bodies rather than aiming for euglycemia (6, 24). Intravenous administration of insulin regular mixed in NaCl 0.9% or D5W as a continuous infusion or intravenous glulisine insulin can be used for the treatment of DKA (2, 25, 26). Standard insulin dilution (1 unit/ml) is commonly used in DKA; however, a more concentrated dilution (16 units/ml) can be used if needed (25,).Insulin can also be used as frequent subcutaneous or intramuscular injections for the treatment of DKA in mild-moderate DKA patients (6, 24). However, a continuous intravenous insulin regimen is preferred over subcutaneous insulin for DKA management overall due to its short half-life, fast onset, and easy titration (6, 24). The use of basal insulin analogs in conjunction with regular insulin infusions may speed up the resolution of DKA and minimize rebound hyperglycemia events, resulting in less ICU length of stay and less healthcare cost (6, 24).

Insulin is currently recommended as a continuous infusion at 0.14 units/kg/hr without a loading dose (LD) (27, 28). Insulin loading dose has been linked to increasing the risk of cerebral edema and worsening shock (29). Thus, insulin loading dose should be avoided at the beginning of therapy (29). However, an insulin loading dose of 0.07–0.1 units/kg over 5 minutes while the patient is receiving a low dose continuous infusion at a rate of 0.07-0.1 units/kg/hr might be utilized to achieve target BG and anion gap (27, 28). Multiple factors must be considered when titrating intravenous insulin continuous infusion (2). The rate of blood glucose reduction, insulin sensitivity, prandial coverage, and NPO status should all be taken into consideration (2). A rapid reduction in BG might be harmful and linked to cerebral edema (2). There are specific characteristics that put patients at higher insulin sensitivity, such as elderly, renal dysfunction, low daily insulin outpatient requirement (< 0.5 U/kg/day) (2). Moreover, the insulin infusion rate can be increased based on BG around major meals time and can be continued at a higher rate for 1-2 hours following any major meal (2). Lastly, it is necessary to monitor BG among NPO patients closely. Maintenance fluid should be combined with 5% dextrose once BG reaches < 250 mg/dL (2).

Randomized clinical trials compared the two strategies and found no difference (27, 28). Intravenous LD insulin administration has been associated with an increased risk of cerebral edema (27, 28). An acceptable alternative for patients with mild to moderate DKA could be a bolus of 0.2 units/kg of subcutaneous rapid-acting insulin is given at the start of treatment, followed by 0.1–0.2 units/kg every 1–3 hours until the BG concentration falls below 250 mg/dL (30, 31). During treatment, the recommended target rate for BG decrease is 50-75 mg/dL/hr for patients with normal renal function (30, 31). Insulin dosing is recommended to be reduced by 50% to reduce the risk of hypoglycemia among patients with kidney disease; however, it should be continued until the resolution of ketoacidosis while maintaining euglycemia (BG: 140 – 180 mg/dL) (30, 31). Patients with end-stage renal disease (ESRD) and acute kidney injury (AKI) are considered a high-risk category that necessitates extra care (32, 33). To avoid rapid increases in osmolality and hypoglycemia in these patients; it is recommended that insulin infusions begin at 0.05–0.07unit/kg/hr with close BG monitoring (32, 33). Dosing can be reduced further or stopped if needed; a study used BG < 120 mg/dL as a cut-off in patients with AKI (32, 33).

Insulin transition from intravenous to a subcutaneous route is essential upon the resolution of DKA, which includes a BG <200 mg/dl and two of the following criteria: a serum bicarbonate level ≥15 mEq/l, a venous pH >7.3, a calculated anion gap ≤12 mEq/l, and the acceptance of oral dietary intake (6, 24). Subcutaneous insulin should overlap with intravenous insulin for at least 30-60 minutes before its discontinuation to ensure the optimal transition of care (6, 24). The Joint British Diabetes Societies guideline for the management of diabetic ketoacidosis” recommends continuation of long-acting insulin analog during the initial management of DKA because it provides background insulin when the intravenous insulin is discontinued” (6, 24). A transition to subcutaneous long-acting insulin in addition to ultra-short acting insulin such as glargine and glulisine after resolution of DKA may result in reduced hypoglycemic events compared to other basal bolus regimens such as NPH insulin and insulin regular (24, 25). For newly diagnosed insulin-dependent diabetes patients, subcutaneous insulin may be started at a dose of 0.5-0.7 units/kg/day (24, 25). The transition process in patients who were previously using insulin or antidiabetic agents before to DKA admission is still unclear (24, 25). In ICU settings, clinicians tend to hold all oral antidiabetic agents and rely on insulin regimens for in-patient management given the shorter half-life of insulin and its predictability (24, 25). This could potentially be an area for further investigation on the transition process and its implication on patient outcomes (24, 25). Insulin sequestering to plastic IV tubing has been described, resulting in insulin wasting and dose inaccuracy (34, 35). Flushing the IV tube with a priming fluid of 20 mL is adequate to minimize the insulin losses to IV tube (34, 35).

## Electrolytes Management

### Potassium Therapy

Patients with hyperglycemic crisiss are at a higher risk of developing hypokalemia due to multifactorial process (1, 29). Insulin therapy, correction of acidosis, and hydration all together lead to the development of hypokalemia (1, 29). Additionally, volume depletion seen with hyperglycemic crisis leads to secondary hyperaldosteronism, which exacerbates hypokalemia by enhancing urinary potassium excretion (1, 29).

Serum potassium level should be obtained immediately upon presentation and prior to initiating insulin therapy (1, 29). Serum potassium level should be maintained within 4 –5 mEq/l (1, 29). Potassium replacement is required regardless of the baseline serum potassium level due to hydration and insulin therapy, except among renal failure patients (1, 29). It is suggested to administer 20 –30 mEq potassium in each liter of intravenous fluid to keep a serum potassium concentration within the normal range (1, 29). Fluid and insulin therapy could be started without potassium replacement if baseline serum potassium is > 5.2 mEq/l (1). If baseline serum potassium level is < 3.3 mEq/l, insulin therapy should be delayed until potassium replacement is completed and potassium level is > 3.3 mEq/l (1, 29).

### Phosphate Therapy

In addition to possible hypokalemia, patients with the hyperglycemic crisis could present with hypophosphatemia (1, 29). Osmotic diuresis during hyperglycemic crisis increases the urinary phosphate excretion, and insulin therapy enhances intracellular phosphate shift (1, 29). Phosphate replacement is not a fundamental part of hyperglycemic crisis management, given the lack of evidence of clinical benefit (1, 29, 36). However, it is recommended to replate serum phosphate level of < 1 mg/dL to avoid severe hypophosphatemia symptoms (cardiac muscle weakness, skeletal muscle weakness, respiratory depression, seizure, and altered mental state) (1, 29). When repletion is indicated, 20–30 mEq/l potassium phosphate could be given (1, 29). A special consideration with phosphate administration is the secondary hypocalcemia (1, 29, 36).

### Bicarbonate Therapy

Acidemia associated with DKA results from the overproduction of ketoacids, generated from the haptic metabolism of free fatty acids. This hepatic metabolism occurs as a result of insulin resistance and an increase in the counterregulatory hormones contributing to the pathophysiology of DKA (37, 38). Tissue acidosis could lead to impaired myocardial contractility, systemic vasodilatation, inhibition of glucose utilization by insulin, and lowering the levels of 2,3-diphosphoglycerate (2,3-DPG) in erythrocytes (37–39). Sodium bicarbonate decreases the hemoglobin-oxygen affinity leading to tissue hypoxia; moreover, it is associated with hypernatremia, hypocalcemia, hypokalemia, hypercapnia, prolonged QTc interval, intracellular acidosis, and metabolic alkalosis (39, 40).

The use of adjuvant sodium bicarbonate in the setting of DKA consistently shows a lack of clinical benefit and should be prescribed on a case-by-case basis. The addition of bicarbonate therapy in severe DKA (defined as a pH of < 6.9) was recommended in ADA hyperglycemic crises guidelines (6). Although this recommendation was not supported by solid evidence; many clinicians adopt the practice to avoid the unwanted side effect of severe metabolic acidosis. Furthermore, life-threatening hyperkalemia (>6.5-7 mEq/l) is considered an indication for bicarbonate therapy (41, 42). Sodium bicarbonate moves potassium intracellularly, however, clinical benefit is uncertain, and the use is controversial (41, 42). If Sodium bicarbonate is indicated due to life-threatening hyperkalemia, 1 mL/kg bolus dose of 8.4% solution or 50-100 mEq sodium bicarbonate in 1 L of appropriate IV solution to be given once until pH increases to >6.9 (42).

## Complication of Hyperglycemic Crisis

Prompt therapy for patients with hyperglycemic crisis is essential in reducing morbidity and mortality (6, 43). If not treated or treated ineffectively, the prognosis can include serious complications such as seizures, organ failures, coma, and death (6, 43). Mortality in the first 48–72 hours occurs in 50% of the hyperglycemic crisis cases due to precipitating cause, hypo or hyperkalemia, and cerebral edema (43). When treatment is delayed, the overall mortality rate of HHS is higher than that of DKA, especially in older patients. This difference in prognoses was comparable when patients were matched for age (43).

In DKA, prolonged hypotension can lead to acute myocardial and bowel infarction (6, 44). The kidney plays a vital role in normalizing massive pH and electrolyte abnormalities (6, 44). Patients with prior kidney dysfunction or patients who developed end-stage chronic kidney disease worsen the prognosis considerably (6, 44). In HHS, severe dehydration may predispose the patient to complications such as myocardial infarction, stroke, pulmonary embolism, mesenteric vein thrombosis, and disseminated intravascular coagulation (6, 44). When compared to other acute medical conditions, data from 2859 hospitalized patients with HHS showed comparable risk of in-hospital venous thromboembolism (VTE) to sepsis [hazard ratio (HR) = 16.3; 95% confidence interval (CI): 10-25] vs sepsis (HR = 19.3; 95% CI: 13-29) (45). The VTE risk was higher than diabetic patients without hyperglycemic crisis or diabetic acidosis patients (45).

Management of hyperglycemic crisis may also be associated with significant complications include electrolyte abnormalities, hypoglycemia, and cerebral edema (7). Electrolyte abnormalities include hypo or hyperkalemia, and hypoglycemia occurred in 25% of the patients treated for hyperglycemic crisis even when standardized protocols were in use (4, 5). This is due to the use of insulin and fluid replacement therapy (4, 5). Therefore, frequent electrolytes and blood glucose concentrations monitoring are essential while insulin infusions and fluid replacements are continued (4, 5).

Cerebral edema is a rare but severe complication in children and adolescents and rarely affects adult patients older than 28 (7). This could be due to the lack of cerebral autoregulation, presentation with more severe acidosis and dehydration among children and adolescents (46). The exact mechanism of cerebral edema development is unknown. Some reports suggest that the risk of cerebral edema during hyperglycemic crisis management might be induced by rapid hydration, especially in the pediatric population. However, a recent multicenter study for 1255 children with DKA who were randomized to receive isotonic versus hypotonic sodium IV fluid with different infusions rates did not show a difference in neurological outcomes (47). Maintaining blood glucose concentration above 250-300 mg/dL for a few hours and avoiding serum osmolality drop by > 3mOsm/kg/hour in patients with hyperglycemic crisis has been suggested as preventive measures for cerebral edema (7, 48). Early identification and prompt therapy with mannitol or hypertonic saline can prevent neurological deterioration from DKA management (7, 48). Furthermore, higher blood urea nitrogen (BUN) and sodium concentrations have been identified as cerebral edema risk factors (46). Thus, careful hydration with close electrolytes and BUN is recommended (46).

Other serious complications of hyperglycemic crisis may include transient AKI, pulmonary edema in patients with congestive heart failure, myocardial infarction, a rise in pancreatic enzymes with or without acute pancreatitis, cardiomyopathy, rhabdomyolysis in patients presented with severe dehydration (7, 24).

## Author Contributions

All authors have contributed equally in writing, organizing, and reviewing this publication.

## Conflict of Interest

The authors declare that the research was conducted in the absence of any commercial or financial relationships that could be construed as a potential conflict of interest.

## Publisher’s Note

All claims expressed in this article are solely those of the authors and do not necessarily represent those of their affiliated organizations, or those of the publisher, the editors and the reviewers. Any product that may be evaluated in this article, or claim that may be made by its manufacturer, is not guaranteed or endorsed by the publisher.
